# Algorithms to anonymize structured medical and healthcare data: A systematic review

**DOI:** 10.3389/fbinf.2022.984807

**Published:** 2022-12-22

**Authors:** Ali Sepas, Ali Haider Bangash, Omar Alraoui, Khaled El Emam, Alaa El-Hussuna

**Affiliations:** ^1^ Open Source Research Collaboration, Aalborg, Denmark; ^2^ Department of Materials and Production, Aalborg University, Aalborg, Denmark; ^3^ STMU Shifa College of Medicine, Islamabad, Pakistan; ^4^ Department of Health Science and Technology, Aalborg University, Aalborg, Denmark; ^5^ Canada Research Chair in Medical AI, University of Ottawa, Ottawa, ON, Canada

**Keywords:** anonymization, de-identification, medical health data, reidentification, electronic health records

## Abstract

**Introduction:** With many anonymization algorithms developed for structured medical health data (SMHD) in the last decade, our systematic review provides a comprehensive bird’s eye view of algorithms for SMHD anonymization.

**Methods:** This systematic review was conducted according to the recommendations in the Cochrane Handbook for Reviews of Interventions and reported according to the Preferred Reporting Items for Systematic Reviews and Meta-Analyses (PRISMA). Eligible articles from the PubMed, ACM digital library, Medline, IEEE, Embase, Web of Science Collection, Scopus, ProQuest Dissertation, and Theses Global databases were identified through systematic searches. The following parameters were extracted from the eligible studies: author, year of publication, sample size, and relevant algorithms and/or software applied to anonymize SMHD, along with the summary of outcomes.

**Results:** Among 1,804 initial hits, the present study considered 63 records including research articles, reviews, and books. Seventy five evaluated the anonymization of demographic data, 18 assessed diagnosis codes, and 3 assessed genomic data. One of the most common approaches was k-anonymity, which was utilized mainly for demographic data, often in combination with another algorithm; e.g., l-diversity. No approaches have yet been developed for protection against membership disclosure attacks on diagnosis codes.

**Conclusion:** This study reviewed and categorized different anonymization approaches for MHD according to the anonymized data types (demographics, diagnosis codes, and genomic data). Further research is needed to develop more efficient algorithms for the anonymization of diagnosis codes and genomic data. The risk of reidentification can be minimized with adequate application of the addressed anonymization approaches.

**Systematic Review Registration**: [http://www.crd.york.ac.uk/prospero], identifier [CRD42021228200].

## Introduction

Over the past two decades, increasing medical health data (MHD) have been collected for secondary purposes such as medical research. MHD contains information such as patient demographics, diagnostics, medication history, and, in some cases, family history. MHD is normally stored in databases available to medical researchers (Gkoulalas-Divanis and Loukides, 2015). While these databases allow researchers to research epidemiology, novel treatment quality, register-based cohort studies, etc. (Gkoulalas-Divanis and Loukides, 2015), they have also increased the risk of reidentification (RR) attack (El Emam et al., 2011). A systematic review by Khaled El Imam and colleagues revealed that 34% of reidentification attacks on medical data were successful (El Emam et al., 2011). Although this study was limited to datasets with relatively small sample sizes, RR is clearly a potentially significant threat (El Emam et al., 2011). To minimize the risk of reidentification due to systematic cyber assaults on MHD, researchers have developed sophisticated techniques and algorithms to anonymize data such that the data can be used for secondary purposes while simultaneously maintaining patient anonymity (Langarizadeh et al., 2018). If data are anonymized sufficiently in compliance with ethical guidelines, written patient consent is not required to utilize their data for secondary purposes; thus, the risk of bias due to a consensus from a fraction of patients and not the entire patient population, is eliminated (El Emam and Arbuckle, 2014). What makes anonymization quite tedious is the delicate balance required between data utility and privacy (El Emam and Arbuckle, 2014). If the data are anonymized to such an extent that they provide no beneficial information about patients, the data are rendered useless; conversely, if the data utility is high, the risk of reidentification grows substantially (Sánchez et al., 2014). One approach to anonymization is Datafly, which applies information generalization, insertion, substitution, and removal to deidentify data (Sweeney, 1998). Another widely utilized deidentification method is optimal lattice anonymization (OLA), which utilizes the k-anonymity method and primarily deidentifies quasi-identifiers (El Emam et al., 2009). A relatively novel anonymization approach is Utility-Preserving Anonymization for Privacy Preserving Data Publishing (PPDP), which also applies the k-anonymity technique and comprises three parts: a utility-preserving model, counterfeit record insertion, and a catalogue of counterfeit records (Sánchez et al., 2014). Although many methods have been suggested, all have strengths and limitations. Moreover, it is not clear, how these different methods compare and which approaches are most suitable for achieving anonymization for a specific purpose.

Therefore, this systematic review aimed to analyze the strengths and weaknesses regarding the RR and data utility of algorithms and software that anonymize structured MHD. As a secondary goal, this study aimed to provide medical health researchers and personnel an opportunity to find and utilize the most suitable algorithm/software for their specific goal(s), by giving an overview of currently available anonymization approaches for structured MHD.

## Methods

This systematic review was conducted according to a pre-defined study protocol. The review was registered in the International Prospective Register for Systematic Reviews (PROSPERO, http://www.crd.york.ac.uk/prospero, reg. no. CRD42021228200) and was conducted according to the Preferred Reporting Items for Systematic Reviews and Meta-Analyses (PRISMA) guidelines for systematic reviews (Page et al., 2021).

### Search strategy

The PubMed, ACM digital library, Medline, IEEE, Embase, Web of Science Collection and Scopus, ProQuest Dissertation, and Theses Global databases were searched systematically. The systematic search terms were discussed with a librarian from the University of Aalborg, Denmark, to ensure that all relevant keywords were included. Additionally, a manual search in the following journals was conducted: *Studies in Health Technology and Informatics*, the *International Journal of e-Healthcare Information Systems*, and the *Journal of Biomedical Informatics* using the search terms “anonymization of medical health data” or “anonymization”. Moreover, manual searches in the reference lists of papers on this topic, contact with experts in bioinformatics, and a campaign using the Twitter and LinkedIn accounts of #OpenSourceResearch collaboration (Open Source Research Organisation, 2021) were used to collect data about any other algorithms/software to ensure that the overview of the subject was as complete as possible. The following keywords were utilized in the systematic search:

- Deidentifi* OR Depersonali* OR Anonymi*
AND
- Medical Health data OR Medical Health records OR Electronic health data OR Electronic medical records OR Digital health records OR Digital medical data,
AND
- Data utility, Data usefulness

### Inclusion and exclusion criteria

#### Inclusion criteria

1. Original studies, reviews, and books about anonymization (de-identification) of structured and/or semi-structured medical data for secondary usage.
2. Studies about anonymized medical records that performed assessments of the risk of reidentification and data utility.
3. Studies that applied or investigated de-identification methods and relevant algorithms to anonymize medical data and assessed the risk of reidentification and data utility.

#### Exclusion criteria

1. To provide the most up-to-date review, studies published before 2000 were excluded. Studies for which the full text was not available were also excluded.
2. Newspaper articles, conference abstracts, and letters to editors were also excluded.

### Screening and data extraction

Two researchers (A.S and O.A) independently conducted the screening using the systematic review software Rayyan (Ouzzani et al., 2016). Any disagreements in exclusion or inclusion were resolved by discussion or the involvement of the senior author (A.E). The following parameters were extracted from eligible studies: author, year of publication, sample size, relevant algorithms and computer programs applied to anonymize MHD, and a summary of outcomes. Data extraction was independently conducted by two authors (A.S and O.A. Disagreements were resolved by discussion or the involvement of the senior author (A.E).

## Results

**FIGURE 1:**
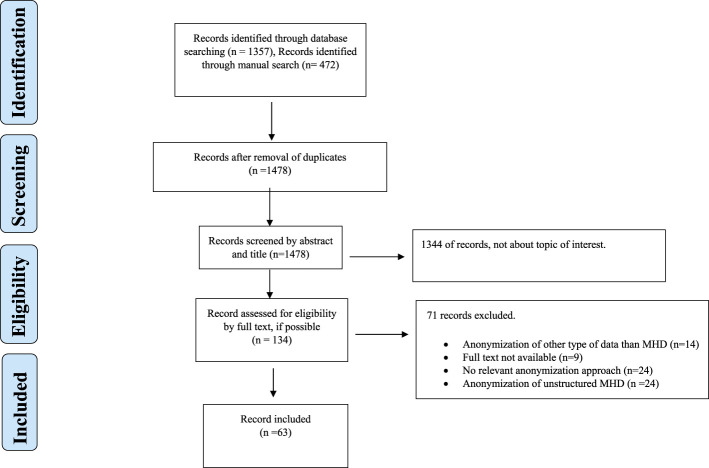
Flowchart.

The systematic search and manual search identified a total of 1,804 records. Figure 1 shows the PRISMA flowchart. In the initial phase of screening by title and abstract, 1,478 records did not meet the inclusion criteria. Thus, 134 records were assessed for eligibility by full-text screening. A total of 63 records were included in the qualitative analysis (Figure 1), comprising 53 research articles, 8 reviews, and 2 books.

The results suggested that anonymization is most widely applied for protection against identity disclosure, primarily Multi-Sensitive (k, θ*)-anonymity (Liu et al., 2021), with θ* denoting different sensitive values, produced anonymized datasets with low levels of information loss and consistently negligible RR for different values of k and θ* (Liu et al., 2021). Supplementary Table S1 provides a detailed summary of the relevant findings from each record. We divided the different anonymization approaches into three categories (anonymization of demographics, diagnosis codes and genomic data) and three sub-categories based on the attack type that they sought to minimize (identity, membership, and attribute disclosures). Tables 1, 2 provide an overview of the different approaches.

**TABLE 1:** Algorithms for the anonymization of structured healthcare data pertinent to demographic data.

	Privacy models	Study number
Attack model	Demographics	
Identity disclosure	k-Minimal generalization Gkoulalas-Divanis et al. (2014)	55
	OLA El Emam et al. (2009)	7
	Incognito Gkoulalas-Divanis et al. (2014)	55
	Genetic Gkoulalas-Divanis et al. (2014)	55
	Mondrian Gkoulalas-Divanis et al. (2014)	55
	TDS Gkoulalas-Divanis et al. (2014)	55
	Greedy Gkoulalas-Divanis et al. (2014)	55
	k-member Gkoulalas-Divanis et al. (2014)	55
	KACA Gkoulalas-Divanis et al. (2014)	55
	Agglomerative Gkoulalas-Divanis et al. (2014)	55
	(k,k)-Anonymizer Gkoulalas-Divanis et al. (2014)	55
	Hilb Gkoulalas-Divanis et al. (2014)	55
	iDist Gkoulalas-Divanis et al. (2014)	55
	MDAV Gkoulalas-Divanis et al. (2014)	55
	CBFS Gkoulalas-Divanis et al. (2014)	55
	LSD Mondrian Gkoulalas-Divanis et al. (2014)	55
	NNG Gkoulalas-Divanis et al. (2014)	55
	r-Gather Aggarwal et al. (2010)	1
	Reliability enhancing software in ARX Bild et al. (2020)	3
	Anonymization of multiple sensitive attributes Chester et al. (2020)	4
	Chrononymization Cimino (2012)	5
	Greedy grouping algorithm Cormode et al. (2010)	6
	OLA El Emam et al. (2009)	8
	k-anonymity and l-diversity based anonymizer Gardner and Xiong (2008)	12
	3-anonymity level using ARX Gentili et al. (2017)	13
	Rare disease anonymization using HIPAA safe harbor Gow et al. (2020)	14
	Objective based anonymization in according to HIPAA rules Jung et al. (2018)	19
	Globally optimal algorithm, can be combined with k-anonymity, l-diversity, t-closeness, δ -presence, or many other methods Kohlmayer et al. (2014)	25
	Generalization and Suppression in ARX Kohlmayer et al. (2015)	26
	Generalization with prevention of overgeneralization Lee et al. (2017)	27
	Counterfeit insertion Lee et al. (2017)	27
	Multi-Sensitive (k, θ*)-anonymity Lin et al. (2016)	28
	Clustering by greedy algorithm and k-anonymization Loukides and Jianhua (2006)	33
	Anonymization according to Safe harbor policy and GenEth disclosure policy Malin et al. (2011)	35
	SDC Martínez et al. (2013)	36
	LKC-privac Mohammed et al. (2009)	38
	SRLA Mohapatra and Patra (2019)	39
	Anonymization with strategies like data swapping, value suppression, generalization, micro aggregation, and noise addition Pika et al. (2020)	41
	De-identification shared task guidelines to longitudinal medical records Stubbs and Uzuner (2014)	45
	HIPAA anonymization rules Tucker et al. (2016)	49
	k-anonymity extension by generalization Ye and Chen (2011)	51
	k-anonymity combined with l-diversity Yoo et al. (2012)	52
	Swapping data anonymization method36	15
	k-anonymity combined with generalization followed by suppression Mawji et al. (2022)	37
	l-diversity slicing approach Onesimu et al. (2022)	40
	Sequential noise addition to event dates k-anonymity with local suppression Templ et al. (2022)	48
Membership disclosure		
	SPALM Gkoulalas-Divanis et al. (2014)	55
	MPALM Gkoulalas-Divanis et al. (2014)	55
	SFALM Gkoulalas-Divanis et al. (2014)	55
	Globally optimal algorithm, can be combined with k-anonymity, l-diversity, t-closeness, δ -presence, or many other methods Gardner and Xiong (2008)	25
	l-diversity slicing approach Onesimu et al. (2022)	40
Attribute disclosure		
	Incognito with l-diversity Gkoulalas-Divanis et al. (2014)	55
	Incognito with t-closeness Gkoulalas-Divanis et al. (2014)	55
	Incognito with (a,k)-anonymity Gkoulalas-Divanis et al. (2014)	55
	p-Sensitive k-anonymity Gkoulalas-Divanis et al. (2014)	55
	Mondrian with l-diversity Gkoulalas-Divanis et al. (2014)	55
	Mondrian with t-closeness Gkoulalas-Divanis et al. (2014)	55
	Top down Gkoulalas-Divanis et al. (2014)	55
	Greedy algorithm Gkoulalas-Divanis et al. (2014)	55
	Hilb with l-diversity Gkoulalas-Divanis et al. (2014)	55
	iDist with l-diversity Gkoulalas-Divanis et al. (2014)	55
	Anatomize Gkoulalas-Divanis et al. (2014)	55
	Delay free anonymization Kim et al. (2014)	23
	Global generalization, local generalization, and bucketization Kim et al. (2017)	24
	Globally optimal algorithm, can be combined with k-anonymity, l-diversity, t-closeness, δ -presence, or many other methods Kohlmayer et al. (2014)	25
	Multi-Sensitive (k, θ*)-anonymity Lin et al. (2016)	28
	HIPAA safe harbor for same disease data (generalization operation utilized) Lin et al. (2016)	29
	LKC-privacy Mohammed et al. (2009)	38
	Closed l-diversification Hsiao et al. (2019)	17
	k-anonymity and l-diversity based anonymizer Gardner and Xiong (2008)	12
	Pseudonymization Somolinos et al. (2015)	44
	k-anonymity combined with l-diversity Yoo et al. (2012)	52
	Combining k-anonymity, l-diversity, and t-closeness Aminifar et al. (2021)	2
	Constraint-based k-means clustering Liu et al. (2021)	30
	l-diversity slicing approach Onesimu et al. (2022)	40

**TABLE 2:** Algorithms for the anonymization of structured healthcare data pertinent to diagnosis codes and genomic data.

	Privacy models	Study number		Study number
Attack model	Diagnosis codes		Genomics data	
Identity disclosure	Combinations Suppression Algorithm Aggarwal et al. (2010)	20	CBA Loukides et al. (2010a)	32
	Clustering based anonymizer (CBA) Bild et al. (2020)	32	∈-differentially private mechanism by adopting the *Laplace mechanism* Yu and Ji (2014)	53
	UGACLIP Gkoulalas-Divanis et al. (2014)	55	∈-differentially private mechanism by adopting the *exponential mechanism* Yu and Ji (2014)	53
	CBA Gkoulalas-Divanis et al. (2014)	55		
	UAR Gkoulalas-Divanis et al. (2014)	55		
	*Apriori* Gkoulalas-Divanis et al. (2014)	55		
	LRA Gkoulalas-Divanis et al. (2014)	55		
	VPA Gkoulalas-Divanis et al. (2014)	55		
	mHgHs Gkoulalas-Divanis et al. (2014)	55		
	Recursive partition Gkoulalas-Divanis et al. (2014)	55		
	k-means clustering Lin et al. (2016)	11		
	Anonymization by “dissociation” with application of -anonymity (Loukides et al., 2014)	34		
Attribute disclosure				
	Greedy Gkoulalas-Divanis et al. (2014)	55		
	Suppress control Gkoulalas-Divanis et al. (2014)	55		
	TDControl Gkoulalas-Divanis et al. (2014)	55		
	RBAT Gkoulalas-Divanis et al. (2014)	55		
	Tree-based Gkoulalas-Divanis et al. (2014)	55		
	Sample-based Gkoulalas-Divanis et al. (2014)	55		

### Anonymization of demographic data

A total of 75 algorithms/software were found for the anonymization of demographic data. Some of these approaches were studied in detail by Gkoulalas-Divanis et al. (2014), a brief summary of which is shown in Supplementary Table S1. Forty six approaches were developed for protection against identity disclosure, 5 against membership disclosure, and 24 against attribute disclosure.

### Methods against identity disclosure

Identity disclosure is the linkage of an individual or a group of individuals to an entry or a few entries in the dataset. This allows the attacker to obtain highly sensitive data about the exposed individuals. Some of the main approaches are micro aggregation (Domingo-Ferrer and Mateo-Sanz, 2002), generalization (Samarati, 2001), and suppression (Samarati, 2001); however, new approaches such as chrononymization (Cimino, 2012) have also been incorporated. Many of the approaches utilize k-anonymity in combination with other methods to improve the performance, such as Multi-Sensitive (k, θ*)-anonymity (Lin et al., 2016), clustering by greedy algorithm and k-anonymization (Loukides and Jianhua, 2006), and k-anonymity combined with l-diversity (Yoo et al., 2012). Similarly, a delicate balance between privacy protection and data utility was achieved by combining clustering by greedy algorithm and k-anonymization (Loukides and Jianhua, 2006). This algorithm provided better overall data utility than Mondrian; however, the data protection provided by Mondrian was better (Loukides and Jianhua, 2006). The combination of l-diversity and k-anonymity reduced information loss compared to l-diversity and conditional entropy (Yoo et al., 2012). k-anonymity has also been extended by generalization (Ye and Chen, 2011) which showed overall better performance than incognito and Mondrian in terms of lower data distortion with increasing k values, smaller information loss, and a linear decrease of information loss with increasing k^34^. Another approach to counter the issue of overgeneralization is the h-ceiling, in combination with k-anonymity, this method showed a significant reduction in information loss compared to k-anonymity alone. Furthermore, the reconstruction error (RE) was also reduced, the lowest level of information loss was achieved with h = 0.25 and the smallest RE with h = 0.35. Thus, overall, it was possible to prevent overgeneralization (Lee et al., 2017). Suppression was also applied in ARX software, which showed the lowest level of increase in data utility with a suppression limit of 5%; however, different utility metrics yielded different results (Cimino, 2012). Chrononymization of a single test result could hinder the risk of reidentification, but overall, this approach did not provide sufficient protection against RR (Cimino, 2012).

### Methods against membership disclosure

Membership disclosure allows an attacker to determine whether data about a particular individual is contained in a dataset. Protection against this type of attack is more challenging than identity disclosure; consequently, only a handful of approaches have been developed to protect against this type of attack, including SPALM, MPALM, SFALM (Gkoulalas-Divanis et al., 2014), and a globally optimal approach that can be combined with l-diversity, t-closeness, δ -presence, or other methods (Mohammed et al., 2009). Most of the existing algorithms share commonalities with those designed for protection against identity disclosure, such as quasi-identifier transformation and heuristic strategies (Gkoulalas-Divanis et al., 2014). SPALM and MPALM transform quasi-identifiers by generalization and attempt to satisfy δ -presence, while simultaneously minimizing information loss (Nergiz et al., 2007). SPALM generalizes all quasi-identifiers of a similar type in the same way, such as generalizing English as an ethnicity to British. MPALM generalizes based on context; for instance, English to British in one context and to European in another (Nergiz et al., 2007). SFALM is similar to the previously mentioned approaches but applies c-confident δ -presence; since this approach does not require complete information about the population, it has higher applicability compared to other approaches (Nergiz and Clifton, 2010). The globally optimal approach produced anonymized distributed datasets with information loss ranging between 13% and 87% (Kohlmayer et al., 2014). This approach showed better performance and lower information loss compared to k-anonymity and l-diversity (Kohlmayer et al., 2014).

### Methods against attribute disclosure

This type of attack attempts to link individuals to a particular entry (entries) in a data set. One of the most popular methods of protecting against attribute disclosure is l-diversity. Several approaches have been combined with l-diversity, including combination with k-anonymity combined (Yoo et al., 2012), Incognito (Gkoulalas-Divanis et al., 2014), and Hilb (Gkoulalas-Divanis et al., 2014). The combination of k-anonymity and l-diversity provides anonymized datasets with minimum information loss, and less information loss compared to l-diversity alone. Only t-closeness had less information loss than the proposed method; this approach was slower than Entropy l-diversity and t-closeness. l-diversity combined with Incognito also provided anonymization with sufficient utility (Machanavajjhala, 2006; Gkoulalas-Divanis et al., 2014) Hilb with l-diversity (Gkoulalas-Divanis et al., 2014) showed better performance in terms of execution time and information loss compared to Incognito combined with l-diversity (Ghinita et al., 2007a). The approach had lower information loss than Mondrian but had a slower performance (Ghinita et al., 2007b; Gkoulalas-Divanis et al., 2014). Incognito has also been combined with t-closeness (Loukides et al., 2010b). The t-closeness approach attempted to overcome the limitations of l-diversity by requiring that the distribution of an attribute in any equivalence class be close to the distribution of the attribute in the overall table (Ghinita et al., 2007a). t-closeness separated the information gained by an observer from a released table into two parts related to all populations in the data and specific individuals, with the gain of the second type of information gain limited in this approach (Ghinita et al., 2007a). Among other approaches, including global generalization, local generalization, and bucketization (Ye and Chen, 2011), the highest information loss was observed for global generalization, followed by local generalization, and bucketization, where information loss was negligible (Kim et al., 2017). The best overall performance was achieved by Bucketization (Kim et al., 2017). LKC-privacy was developed for larger datasets and was more suitable for blood transfusion service (BTS) data. LKC-privacy allows data sharing, thus providing higher flexibility for BTS data (Mohammed et al., 2009) and higher overall quality of data than k-anonymity (Yoo et al., 2012). For faster anonymization, delay-free anonymization (DF) was developed, which anonymized a single tuple in 0.037 ms compared to 0.18 ms for the accumulated-based method (ABM-1). Information loss by DF was significantly lower than ABM-1, and the l-diverse data set was preserved with a probability of 1/l^40^. Pseudonymization is also a novel approach that allows researchers to adjust the relevant parameters for optimal results (Somolinos et al., 2015).

### Anonymization of diagnosis codes

The comprehensive systemic search for models of diagnosis code privacy yielded 18 algorithms that aimed to secure diagnosis codes from privacy breaches, unintentional or otherwise. All of these algorithms were related only to identity disclosure. El Emam et al. proposed their Combinations Suppression Algorithm for cases with overlapping combinations of quasi-identifiers and reported less information loss compared to the complete suppression algorithm (Emam et al., 2011). The clustering-based anonymizer (CBA) was presented by Loukides et al. (2010b; Loukides et al. (2010a) for the anonymization of diagnosis codes by clustering and subsequently compared its performance to that of UGACLIP. Comparatively higher satisfaction of utility constraints was reported for CBA with lesser information loss, for the Normalized Certainty Penalty and Average Relative Error (Loukides et al., 2010b). The review by Gkoulalas-Divanis et al. (2014) provided a snapshot of contemporary diagnosis codes privacy algorithms and outlined several key algorithms including, among others, recursive partition, local recoding generalization, and mHgHs. K-means form the basis of a couple of pertinent algorithms related to clustering (Gal et al., 2014) and dissociation anonymization (Gkoulalas-Divanis et al., 2014).

### Anonymization of genomic data

A comprehensive search yielded only three privacy algorithms and explored their applications vis-a-vis genomic data based only on the requirements of identity disclosure. The CBA algorithm not only preserved the genomic information but also exhibited superior anonymization capabilities (Loukides et al., 2010b). Yu and Ji (2014) developed algorithms that respectively extended the Laplace and exponential mechanisms and evaluated c^2^ statistics and Hamming distance scores to consider the algorithmic performance when applied to a set of single-nucleotide polymorphisms (Yu and Ji, 2014). The superiority of the ∈-differentially private mechanism as extended from the exponential mechanism was demonstrated using the Hamming distance as the score function. However, limitations were demonstrated for the Hamming distance, specifically the early plateau of genomic data utility and the effects of the threshold *p*-value on the data utility (Yu and Ji, 2014).

## Discussion

The results of this systematic review demonstrated the feasibility of the anonymization of different types of data such as demographics, diagnosis codes, and genomic data with sufficient levels of protection and utility. The main findings were that for the anonymization of demographics, the combination of classical approaches such as Multi-Sensitive (k, θ*)-anonymity (Lin et al., 2016), extension of k-anonymity with generalization, the combination of k-anonymity with l-diversity (Yoo et al., 2012), and Incognito with l-diversity (Gkoulalas-Divanis et al., 2014) generally provided better data utility and protection than either of methods alone. Issues such as overgeneralization and slow performance were also addressed (Kim et al., 2014; Lee et al., 2017). Moreover, a comparison of some of the algorithms provided researchers an opportunity to select the most suitable anonymization approach for their specific purposes. The findings of this systematic review are consistent with those reported by Langarizadeh et al. (2018) and El Emam and Arbuckle (2014), who concluded that the currently available anonymization approaches provide a delicate balance between data utility and RR for demographic data; however, it is impossible to eliminate RR. Many of the methods are computationally costly, especially for large amounts of data. Pseudonymization was easier to implement for larger data sets and allowed the linkage of data without retaining all identifying characteristics, in contrast to other state-of-the-art approaches.

The potential psychological, financial, and even physical harm to which a patient can be exposed secondary to privacy breaches of diagnosis codes cannot be overstated. Therefore, the optimization of diagnosis code privacy should have paramount significance as the ultimate endpoint for healthcare privacy projects. This snapshot of the diagnosis code privacy-protecting algorithms attempts to reinforce the established considerations among the global healthcare privacy research community including, but not limited, to a heightened recognition of the unambiguous requirement for the development of approaches to optimize statistical analysis capabilities embedded in the information provided by the diagnosis codes with concurrent enhanced suppression of such codes to make it almost impossible for them to be exploited for malicious intent. This may be attained *via* suppressive algorithms that attempt to attenuate utility constraints to a bare minimum. Gkoulalas-Divanis et al. (2014) described the respective pros and cons of different algorithmic models driven by privacy techniques aimed at anonymizing diagnosis codes. Suppression, when employed simultaneously with generalization, provides higher orders of privacy and statistical capabilities compared to those for suppression alone (Gkoulalas-Divanis et al., 2014). Comparisons of bottom-up and top-down heuristic partitioning strategies have demonstrated higher statistical capabilities provided for bottom-up approaches whereas clustering strategies, as those employed by algorithms such as CBA, provide even higher statistical capabilities, although they are computationally expensive (Gkoulalas-Divanis et al., 2014). A strategy to concatenate bottom-up and top-down partitioning strategies has also been reported to optimally provide holistic privacy requirements and concurrently show the superior statistical capabilities provided by diagnosis codes (Gkoulalas-Divanis et al., 2014).

### Limitations and directions for future research

The review has some limitations. First, the included studies used different metrics for the assessment of data utility and risk of reidentification, making comparisons of the two approaches challenging, particularly when different metrics were applied for performance evaluation. A wide variety of methods exist for protection against attribute disclosure and identity disclosure, in contrast to the handful of available approaches for protection against membership disclosure. Future research must address this issue, with greater emphasis on protection against membership disclosure. Although anonymization did not provide any apparent advantages over traditional methods (Cimino, 2012), additional research is required to support these findings and further elaborate on the advantages and shortcomings of anonymization. Similarly, pseudonymization is a relatively novel and unexplored domain that requires further investigation, since some clear benefits of this method have been demonstrated (Tinabo et al., 2009). The present study mainly focused on structured MHD; however, novel methods have been developed to handle medical journals and medical images. Our future work aims to also systematically review these anonymization approaches.

## Conclusion

In summary, this study reviewed different anonymization approaches for MHD and categorized them according to the anonymized data type (demographics, diagnosis codes, and genomic data). The strengths and limitations of algorithms that protect against identity, attribute, and membership disclosure were addressed. Further research is needed to develop more efficient algorithms for the anonymization of diagnosis codes, and genomic data. The less explored approaches such as chrononymization and pseudonymization yielded promising results of interest for further research. The risk of reidentification can be minimized with adequate application of the included anonymization approaches.

## Data Availability

The raw data supporting the conclusions of this article will be made available by the authors, without undue reservation.
