# Supplementary material for: Algorithms to anonymize structured medical and healthcare data: A systematic review
Source: Front Bioinform. 2022 Dec 22;2:984807. doi: 10.3389/fbinf.2022.984807 (PMC9815524; doi:10.3389/fbinf.2022.984807)
Supplement: Supplementary file 1 [file Table1.pdf]

Supplementary Table 1 Characteristics of the included records and related outcomes.

<!--Col Count:8-->Study number	Author (year)	Sample size	Category	Aim	Type of anonymized medical health data	Software and/or algorithm main characteristics	Related outcomes
1	Aggarwal et al., 2006 Aggarwal et al. (2010)	-	Anonymization<br>Data utility	Anonymization of quasi-identifiers by clustering	-	R-Gather technique clustering quasi-identifiers<br><br>Cellular Clustering as a clustering metric	- The first constant-factor approximation algorithm for anonymization<br><br>- Generalization of algorithms, such that ϵ fraction of points remained unclustered<br><br>- Less distortion of data and higher utility than k-anonymity in general
2	Aminifar et al., 2021 Aminifar et al. (2021)	282 complete records	De-identification<br>Data utility	Anonymization that addresses data-, and	Cleveland's processed dataset	Considered k-anonymity, l-diversity, and t-	- Diversity constraint introduction did not affect data utility

<!--Col Count:8-->Study number	Author (year)	Sample size	Category	Aim	Type of anonymized medical health data	Software and/or algorithm main characteristics	Related outcomes
				attribute-linkage attack models		closeness in a unified framework	- F1-score, accuracy, and Matthews Correlation Coefficient (MCC) in anonymized datasets decreased with increasing values of k<br><br>- Ranges for F1-score, accuracy, and MCC were (74%–82%), (76%–82%), and (0.50%–0.63%), respectively<br><br>- Protection against attack types were achieved, while maintaining sufficient data utility

<!--Col Count:8-->Study number	Author (year)	Sample size	Category	Aim	Type of anonymized medical health data	Software and/or algorithm main characteristics	Related outcomes
3	Bild et al., 2020 Bild et al. (2020)	-	Anonymization Data utility	Improvement of anonymized data's reliability	Fictional dataset by researchers	Software integrated into ARX to increase reliability by decreasing the vulnerability from the floating-point operations	- Such integration is reported to be approximately four times faster than the floating-point implementation in ARX.<br><br>- Data utility loss is reported to be negligible in the said implementation in comparison to the floating-point implementation
4	Chester et al., 2020 Chester et al. (2020)	15,830 entries from MEPS 8,000 rows	Anonymization Data utility	Effect of k-anonymity of differential privacy on	Healthcare data from the company MEPS.	k-anonymity Differential-privacy	- Accuracy of k-anonymity deteriorates with higher k

<!--Col Count:8-->Study number	Author (year)	Sample size	Category	Aim	Type of anonymized medical health data	Software and/or algorithm main characteristics	Related outcomes
		from Adult dataset		fairness and utility	Adult dataset, public data set from University of California Irvine (UCI) Machine Learning Repository		- No loss of accuracy for different k values in k-anonymity for de-identified data, similar results for different ϵ values in differential privacy<br>- The fairness measure had much variability for different k- and ϵ values in k-anonymity and differential privacy
5	Climino et al., 2012 Cimino (2012)	20,000 panels	Chrononymization RR assessment	Determination of whether removal of dates from test panels reduces risk of reidentification	Laboratory test panels	Removal of dates from the original data set.	- Ps of a single test result can hinder reidentification<br>- No sufficient reduction of RR due to chrononymization

<!--Col Count:8-->Study number	Author (year)	Sample size	Category	Aim	Type of anonymized medical health data	Software and/or algorithm main characteristics	Related outcomes
6	Cormode et al., 2010 [Cormode et al. (2010)]	-NA	RR	Protection against minimality attacks while Maintaining data utility simultaneously	Fictional dataset	Greedy Grouping (GG) and Randomized Greedy Grouping Method (RGG)	- Data sets protected against minimality attacks with the probability set to *p* = 0.65
			Data utility				- RGG capable of rendering minimality attacks ineffective
							- Minimality attack probability = 1/L in GG method, with utilization of l-diversity measure
7	Dankar et al., 2012	934,025	RR	Estimation of uniqueness as a	3 public data sets	Monte Carlo simulations and utilization of	- Equivalent performance of four estimators for high

<!--Col Count:8-->Study number	Author (year)	Sample size	Category	Aim	Type of anonymized medical health data	Software and/or algorithm main characteristics	Related outcomes
	(Dankar et al., 2012)			direct measure of RR.	3 clinical datasets from hospital registries	Zayatz, slide negative binomial (SNB), the Pitman model, and the µ-Argus as uniqueness estimators for RR prediction.	sampling fraction, median relative bias = 0.22<br>- Lowest median relative bias = 0.013 of the Pitman- and µ-Argus model for low sampling fractions <30%, here highest relative bias from Zayatz and SNB.<br>- Decision rule created for most accurate measurement of RR.

<!--Col Count:8-->Study number	Author (year)	Sample size	Category	Aim	Type of anonymized medical health data	Software and/or algorithm main characteristics	Related outcomes
8	El Emam et al., 2009 El Emam et al. (2009)	-	De-identification	K-anonymity based de-identification approach	Hospital registry datasets	Optimal Lattice Anonymization approach based on k-anonymity	- Higher information loss of Samarati and Datafly compared to OLA as measured by the discernibility and non-uniform entropy metric<br>- Datafly faster than Samarati<br>- OLA significantly faster than Samarati, Datafly, and Incognito<br>- The information loss reported for OLA equal to that reported for Incognito

<!--Col Count:8-->Study number	Author (year)	Sample size	Category	Aim	Type of anonymized medical health data	Software and/or algorithm main characteristics	Related outcomes
9	El Emam et al., 2011 El Emam et al. (2011)	2,375,331 records	De-identification	Public use micro data file (PUMF) from Discharged abstract data (DAD)	2008–2009 DAD, contains administrative, clinical and demographic data on hospital discharges	K-anonymity based anonymization	- At threshold of 0.05, maximum proportion of records with diagnosis codes suppressed was reported to be 20%, which was only 8–9% of DAD.
			Data utility				- At threshold of 0.04, 13.6% suppression of geographical PUMF by complete suppression algorithm was reported to be reduced to 8.4%
10			Anonymization Data utility			Micro aggregation,	- Anonymization with significantly less

<!--Col Count:8-->Study number	Author (year)	Sample size	Category	Aim	Type of anonymized medical health data	Software and/or algorithm main characteristics	Related outcomes
	Gadad et al., 2021 Gadad et al. (2021)			Anonymization with minimal information loss	Fictional dataset by researchers	Permutation, anatomy, and slicing methods	information loss in comparison to generalization and suppression techniques<br>- Preservation of multiple sensitive attributes with minimal information loss<br>- Overcame the background knowledge, linking, and homogeneity attacks
11	Gal et al., 2014 Lin et al. (2016)	26,973	De-identification	Creation of a de-identification approach, accounting for requirements of the data recipient	Data from colon and lung cancer patients	Anonymization by k-means clustering algorithm	- Percentage of coefficients changed significantly between 1.85% and 26%. Lower percentage means a higher utility preservation

<!--Col Count:8-->Study number	Author (year)	Sample size	Category	Aim	Type of anonymized medical health data	Software and/or algorithm main characteristics	Related outcomes
			Data utility				- Linear correlation between data size and performance time except for Two Fixed Reference Points (TFRP) algorithm<br>- For increasing size of data sets, the proposed method much faster than TFRP, but slower than a suppression or generalization utilizing commercial algorithm
12	Gardner et al., 2008 Gardner and Xiong (2008)	100 pathology reports	De-identification	De identification of medical health information	Cancer patients	k-anonymity and l-diversity	- Precision between 0.97 and 1.00. Recall rate and F1 metric ranges between 0.96–1.00 and 0.97–1.00, respectively

<!--Col Count:8-->Study number	Author (year)	Sample size	Category	Aim	Type of anonymized medical health data	Software and/or algorithm main characteristics	Related outcomes
			Data utility				- The anonymization of MHD while maintaining sufficient privacy and maximizing data utility by statistical de-identification approach<br>- The larger the k value, the higher the level of privacy
13	Gentili et al., 2017 Gentili et al. (2017)	11,000 records	Anonymization	Anonymization of high dimensional medical data, assessment of utility and disclosure risk	Hospital survey data	ARX	- Almost a unique data set created by using 6/121 attributes

<!--Col Count:8-->Study number	Author (year)	Sample size	Category	Aim	Type of anonymized medical health data	Software and/or algorithm main characteristics	Related outcomes
			Data utility<br><br>RR				- Disclosure as high as 72.68% when 6 attributes utilized<br><br>- Disclosure risk increased with the number of attributes<br><br>- Information loss on average 6.1%
14	Gow et al., 2020 Gow et al. (2020b)	80 patients	RR assessment	RR estimation of datasets with rare disease patients	Data mining from patient support forums	HIPAA's 'Safe Harbor	- Patients with rare disease conditions deidentified by HIPAA's 'Safe Harbor' were calculated to be at a 75% risk of re-identification

<!--Col Count:8-->Study number	Author (year)	Sample size	Category	Aim	Type of anonymized medical health data	Software and/or algorithm main characteristics	Related outcomes
15	Gunawan et al., 2021 Gunawan et al. (2021)	1,024 records	Anonymization<br>Data utility	Anonymization of prescription data	Retail dataset like prescription dataset	Application of swapping data anonymization method<br><br>Sufficiently similar records are swapped together	- Sensitive item sets were not linked to original data after swapping<br><br>- 175/3,235 distinct items were lost after anonymization<br><br>- Dissimilarity value between original and anonymized data set was 0.00057<br><br>- Information loss was 0.669 as measured by Kulback-Liebler divergence (KL-divergence)

<!--Col Count:8-->Study number	Author (year)	Sample size	Category	Aim	Type of anonymized medical health data	Software and/or algorithm main characteristics	Related outcomes
							- Sufficient degree of data utility for application in data mining
16	Heatherly et al., 2016 Heatherly et al. (2016)	400 patients	Anonymization Data utility assessment	De-identification of data from multiple medical centers	Clinical data from 3 centers	State of the art k-anonymity model utilized	- Feasible data sharing between multiple centers with preservation of utility and privacy
						Hierarchy of ICD9 codes utilized	- Possible to release larger amounts of data than previously believed
							- Reduction of anonymization regions 15%–0.5%
17	Hsiao et al., 2019 Hsiao et al. (2019)	-	Anonymization	Anonymization of data with missing values	FDA Adverse Event Reporting	I-diversification approach	- Reduction of privacy leakage and sustained data utility

<!--Col Count:8-->Study number	Author (year)	Sample size	Category	Aim	Type of anonymized medical health data	Software and/or algorithm main characteristics	Related outcomes
			Data utility		System (FAERS)<br><br>Public dataset regarding adverse events of drugs	3 approaches incorporated to account for missing values: deletion, imputation, and preservation	- Data with worst utility generated by deletion strategy as measured by normalized information loss<br><br>- Preservation strategy most effective in maximizing data utility
18	Jayapradha and Prakash 2021	120,400 records	Anonymization	Multi-valued record anonymization of a patient	Medical records. User records	Vertical partitioning k-anonymity for	- Satisfactory performance for multi-records

<!--Col Count:8-->Study number	Author (year)	Sample size	Category	Aim	Type of anonymized medical health data	Software and/or algorithm main characteristics	Related outcomes
						quasi-identifier bucket (QIAB)	
			Data utility			(k,l)-diversity applied for multi sensitive attribute bucket (IMSB)	- Successful prevention of attacks by sensitive attribute linking
							- Higher classification utility than RMR algorithm for different values of k
19	Jung et al., 2018 Jung et al. (2018)	137 clinical data set columns	Anonymization	Creation of highly secure de-identified personal health information (PHI)	Data from cancer patients	De-identification in accordance with HIPAA rules	- Assessment of overall data quality for secondary analysis by a new scale, based on an average value, calculated from the following parameters: possibility of data linkage,

<!--Col Count:8-->Study number	Author (year)	Sample size	Category	Aim	Type of anonymized medical health data	Software and/or algorithm main characteristics	Related outcomes
							data reidentification and user understanding of data
						Providence of an approach where Quasi identifiers are selected based on objectives for data usage	- Minimization of data loss and maximization of privacy obtained
							- 14.6% and 12.4% of columns had a identifiability score of ≤0.5 and ≤0.75 respectively
20	Kanwal et al., 2021	27,182 records	Anonymization	Anonymization of MHD utilizing many databases	YouTube dataset	“Combinations Suppression Algorithm”:	- 1: M MSA-(p, l)-diversity model efficient with

<!--Col Count:8-->Study number	Author (year)	Sample size	Category	Aim	Type of anonymized medical health data	Software and/or algorithm main characteristics	Related outcomes
	Kanwal et al. (2021)			and multiple attributes		Combination of multiple datasets in to one, masking afterwards and lastly performance of l-diversity for the most vulnerable attributes	significant increase in data utility
		50,000 tuples	Data-utility		Adults dataset		- Zero query error, much better than p + sensitive, k -anonymity, (P,L) Angelization, and 1:M generalization
					Informs dataset: data from patients		- Much smaller execution time than previously explored techniques

<!--Col Count:8-->Study number	Author (year)	Sample size	Category	Aim	Type of anonymized medical health data	Software and/or algorithm main characteristics	Related outcomes
					in medical centers		
21	Khan et al., 2021 Khan et al. (2021)	Up to 50,000 records	Anonymization	Anonymization of quasi-identifiers	Adult dataset	Organization of records by utilizing absolute similarity index	- Privacy was preserved while maintaining data utility
			Data-utility			Data generalization by utilizing global recoding scheme	- Used 18,716 times less storage than Attribute-centric anonymization method
							- Drastic decrease of performance time in comparison to Attribute-centric anonymization method

<!--Col Count:8-->Study number	Author (year)	Sample size	Category	Aim	Type of anonymized medical health data	Software and/or algorithm main characteristics	Related outcomes
22	Khokhar et al., 2014 Khokhar et al. (2014)	45,222 records	De-identification	Cost-benefit analysis of various anonymization approaches	Adult dataset.	K-anonymity	- Quantification of trade-off between data-utility and privacy
			Privacy protection			LKC-privacy	- Analytical cost model developed: can identify the optimal value for publishing data and the damage cost due to privacy breaches
						$\varepsilon -$ differential privacy	- Model applicable to K-anonymity, LKC-privacy, $\varepsilon -$ differential privacy, perturbative and nonperturbative anonymization approaches

<!--Col Count:8-->Study number	Author (year)	Sample size	Category	Aim	Type of anonymized medical health data	Software and/or algorithm main characteristics	Related outcomes
23	Kim et al., 2014 Kim et al. (2014)	149,608	Anonymization	Achieve delay-free anonymization (DF)	Adult data set	Organization of I-diverse data streams in real time	- Anonymization of one tuple 0.037 ms by DF, accumulated based method (ABM-1) 0.18 ms
					National patients' sample	Central components of the new algorithm: late validation and counterfeit generation	- DF considerably faster processing time per tuple than ABM-1 0.09 ms vs 0.8 ms, respectively
							- Significantly less data loss compared to accumulation methods
24	Kim et al., 2017 Kim et al. (2017)	82,561 records	Anonymization	Assessment of anonymization quality	Adult data set	Three algorithms	- Global generalization with highest information average loss metric (LM) =

<!--Col Count:8-->Study number	Author (year)	Sample size	Category	Aim	Type of anonymized medical health data	Software and/or algorithm main characteristics	Related outcomes
							0.35. Local generalization average LM = 0.08 and Bucketization LM negligible
					National patients' sample	Assessed: Global generalization, Local generalization, and Bucketization	- The best performance by Bucketization as measured by average median relative error (point queries) = 0.01%, local generalization = 6.77% and global generalization = 18.90%
							- The overall best performance observed to be that of Bucketization

<!--Col Count:8-->Study number	Author (year)	Sample size	Category	Aim	Type of anonymized medical health data	Software and/or algorithm main characteristics	Related outcomes
25	Kohlmayer et al., 2014 [Kohlmayer et al. (2014)]	1,927,297 records	Anonymization	Anonymization of distributed datasets in the semi honest model	5 different public national datasets	Centralized anonymization algorithm	- Comparison of information loss to two other approaches: anonymize and integrate of horizontally and vertically distributed data applied for k-anonymity and l-diversity
						Globally optimal algorithm combined with either k-anonymity, l-diversity, t-closeness or δ-presence	- Up to 87% reduction in information loss

<!--Col Count:8-->Study number	Author (year)	Sample size	Category	Aim	Type of anonymized medical health data	Software and/or algorithm main characteristics	Related outcomes
							- Less information loss in 75% of cases, when a setup of three parties is compared to a setup of two parties
26	Kohlmayer et al., 2015 Kohlmayer et al. (2015)	1,927,297 records	Anonymization	Anonymization with minimal information loss	Same as Kohlmayer et al. (2014)	Generalization and Suppression	- Considerable increase of data utility for suppression limit of 5%
						Software: ARX system with utilization of AnonBench benchmarking environment	- Lowest data utility increase = 39% for suppression limit of 5%, but data utility percentage highly dependent on the utility metric
							- Anonymized data with acceptable utility level by

<!--Col Count:8-->Study number	Author (year)	Sample size	Category	Aim	Type of anonymized medical health data	Software and/or algorithm main characteristics	Related outcomes
							suppression and generalization
27	Lee et al., 2017 Lee et al. (2017)	15 attributes and 32,561 rows from UCI	Anonymization	A utility preserving anonymization approach for privacy preserving data publishing (PPDP)	Dataset from Health Insurance Review and Assessment service in Korea) HIRA.	k-anonymity, suppression, and generalization	- Significant reduction of information loss by K-anonymity with h-ceiling for an h value of 0.3 as measured by LM.
		1,375,900 records from NPS				h-ceiling introduced for prevention of overgeneralization	- Reconstruction error (RCE) diminished as well by k anonymity with h = 0.3 compared to k-anonymity alone

<!--Col Count:8-->Study number	Author (year)	Sample size	Category	Aim	Type of anonymized medical health data	Software and/or algorithm main characteristics	Related outcomes
							- Data-truthfulness of an anonymized dataset was observed to be preserved
28	Lin et al., 2016 [Lin et al. (2016)]	-	Anonymization Data utility	Anonymization of data from spontaneously reporting systems (SRS)	FAERS datasets	Multi-Sensitive (k, θ*)-anonymity MS(k, θ*)-approach, a hybrid of clustering and greedy methods. Hierarchy-based and free generalization approaches utilized too	- Similar performance by (α, k)-anonymity and MS(k, θ*)
							- Information loss (as measured by normalized information loss (NIL))

<!--Col Count:8-->Study number	Author (year)	Sample size	Category	Aim	Type of anonymized medical health data	Software and/or algorithm main characteristics	Related outcomes
							from algorithms much smaller than multi-sensitive l-diversity approach<br><br>- Anonymization of SRS datasets with sufficient utility and privacy protection achieved
29	Liu et al., 2016 (Liu et al., 2016)	480 patients	Anonymization Disclosure risk assessment	Algorithm for efficient anonymization of same-disease data	Hospital data from patients who received surgery for the same disease	Generalization operation as in k-anonymity	- Application of the same standards to the releasing of all data by HIPAA Safe Harbor leads to overprotection for some and under protection for other data sets

<!--Col Count:8-->Study number	Author (year)	Sample size	Category	Aim	Type of anonymized medical health data	Software and/or algorithm main characteristics	Related outcomes
							- Safe Harbor method is 14 times faster than the proposed method with an equal information loss
30	Liu et al., 2021 [Liu et al. (2021)](#)		Anonymization Data utility	Algorithm for anonymization of socioeconomic data to be utilized for medical research	2017 census tract-level deprivation index dataset	Constraint-based k-means clustering approach	- Lowest overall KL divergence by proposed method, highest by Greedy Algorithm<br>- Better overall data utility than Greedy Algorithm, k-anonymity, and proposed method by HIPAA Safe Harbor<br>- Larger values of k in the proposed method did not

<!--Col Count:8-->Study number	Author (year)	Sample size	Category	Aim	Type of anonymized medical health data	Software and/or algorithm main characteristics	Related outcomes
							consistently lead to better overall utility
31	Loukides et al., 2010a Loukides et al. (2010a)	2,800 patients	Anonymization RR	Risk of reidentification assessment	Medical records from Vanderbilt University Medical Center	Perturbation techniques such as suppression and generalization	- Highest level of privacy with <0.0% of patients being reidentified obtained by generalization followed by suppression with threshold suppression of 25%<br>- High distortion of data by suppression<br>- Suppression of rare codes as required by HIPAA nor generalization able to achieve sufficient privacy

<!--Col Count:8-->Study number	Author (year)	Sample size	Category	Aim	Type of anonymized medical health data	Software and/or algorithm main characteristics	Related outcomes
							for data set while simultaneously maintaining data utility
32	Loukides et al., 2010b Loukides et al. (2010b)	First data set: 5,830 distinct ICD codes	Anonymization	Prevention of reidentification through linkage of genomic data with diagnosis codes	Medical records from Vanderbilt University Medical Center	Clustering based anonymizer (CBA). Linkage of data to at least k patients with potentially identifying diagnosis codes by CBA.	- Satisfaction of more data utility constraints by CBA for k = 5, compared to UGACLIP relative improvement 22.2% and 5.5% for the two data sets respectively
		Second data set					- CBA = UGACLIP in privacy protection

<!--Col Count:8-->Study number	Author (year)	Sample size	Category	Aim	Type of anonymized medical health data	Software and/or algorithm main characteristics	Related outcomes
		305 distinct ICD codes					- CBA more demanding computationally than UGACLIP.
33	Loukides and Shao 2006 [Loukides and Jianhua (2006)]	500 tuples in Adults data set	Data utility Privacy protection	Proposing a clustering based De-identification algorithm that balances privacy protection and data utility	Adult dataset and dataset created by researchers.	Clustering based algorithm with utilization of the metric for k-anonymization	- Delicate balance between privacy protection and data utility achieved by clustering-based anonymization
						Clustering achieved by a greedy algorithm	- New approach ≥ Mondrian in data utility
							- Mondrian ≥ new approach in data protection

<!--Col Count:8-->Study number	Author (year)	Sample size	Category	Aim	Type of anonymized medical health data	Software and/or algorithm main characteristics	Related outcomes
34	Loukides et al., 2014 [Loukides et al. (2014)]	58,302	Anonymization Data utility	Anonymization and increase of data utility	Hospital dataset from patients submitted to elective surgery	Anonymization by “dissociation”	- Specification of diagnosis codes not required
						Application of k^m-anonymity	- Considerably higher data utility than CBA as measured by average relative error (ARE)
							- Faster performance than CBA especially for data with more records
35	MalinTable et al., 2011	18,204 patients	RR	RR assessment of data released in accordance with	Data set from various organizations	Anonymization according to Safe Harbor policy and	- Safe Harbor total RR between 0.01% and 0.19%

<!--Col Count:8-->Study number	Author (year)	Sample size	Category	Aim	Type of anonymized medical health data	Software and/or algorithm main characteristics	Related outcomes
	Malin et al. (2011)			Safe Harbor policy		GenEth disclosure policy	- Total RR of data disclosed in accordance with 10-year GenEth policy between <0.01% and 0.03%
36	Martínez et al., 2013 Martínez et al. (2013)	3,006 records	De-identification	Improvement of data utility	Hospital inpatient data	Improvement of Statistical Disclosure control of non-numerical data. Semantically grounded operators utilized: comparison, aggregation, and sorting. Instead of	- Semantically coherent interpretation of non-numerical attributes achieved by utilization of operators

<!--Col Count:8-->Study number	Author (year)	Sample size	Category	Aim	Type of anonymized medical health data	Software and/or algorithm main characteristics	Related outcomes
						omitting the semantic component of non-numerical data	- Semantic preservation increased significantly<br>- Performance speed constant = approximately 750 s for semantic recoding
37	Mawji et al., 2022 Mawji et al. (2022)	1750 patients	De-identification Data utility	De-identification of identifiers and quasi-identifiers	Hospital inpatient data	k-anonymity combined with generalization followed by suppression	- k-anonymity satisfied with minimal suppression key variables used for modeling

<!--Col Count:8-->Study number	Author (year)	Sample size	Category	Aim	Type of anonymized medical health data	Software and/or algorithm main characteristics	Related outcomes
						R package sdcMicro	- Distribution of response values remained in general close to 1% before and after suppression. Variable integrity remained intact<br>- De-identification of dataset with sufficient level of privacy and data utility was obtained
38	Mohammed et al., 2010 Mohammed et al. (2009)	150,000 records	De-identification	Two anonymization approaches introduced for high dimensional MHD.	Red cross Blood Transfusion Service (BTS) data	LKC-privacy with application of progressive generalization. Possible for users to choose trade-off between privacy and accuracy	- Approach well suited for anonymizing large datasets

<!--Col Count:8-->Study number	Author (year)	Sample size	Category	Aim	Type of anonymized medical health data	Software and/or algorithm main characteristics	Related outcomes
							- The algorithm is more suited to BTS compared to privacy-preserving data mining (PPDM), due to allowance of data sharing, a necessity for BTS, thus higher flexibility and better opportunity for analysis of BTS datasets<br><br>- Better data quality achieved than traditional k-anonymity approach as measured by discernibility ratio
39	Mohapatra et al., 2019 Mohapatra	NA	Hyper anonymization Privacy protection	Development of rank label anonymization	Nursery data from UCI Machine	Sequential rank label	- Higher disclosure risk by rank label anonymization than rank anonymization

<!--Col Count:8-->Study number	Author (year)	Sample size	Category	Aim	Type of anonymized medical health data	Software and/or algorithm main characteristics	Related outcomes
	and Patra (2019)			algorithm and studying of rank-label anonymization attacks	Learning Repository	anonymization (SRLA)	ranging between 50% and 90%. Rank anonymization disclosure risk between 50% and 90%
						Minimization of anonymization cost by finding the correct rank labels sequence	- Better overall performance and lower anonymization cost by SRLA than Greedy Rank Label Anonymization
						A clustering approach utilized to detect the appropriate anonymization sequence	

<!--Col Count:8-->Study number	Author (year)	Sample size	Category	Aim	Type of anonymized medical health data	Software and/or algorithm main characteristics	Related outcomes
40	Onesimu et al., 2022 Onesimu et al. (2022)		Anonymization Data utility	Produce attribute-focused privacy preserving data	Adult-7 and Adult-15 datasets	Fixed-interval approach for protection of numerical attributes	- $\approx 12\%$ less information loss as measured by Normalized Certainty Penalty than Mondrian, (k, k_m)- anonymity, and clustering-based k-anonymity<br>- Information loss consistently small with increasing k-values<br>- Faster computation time than Mondrian, and the approach IACk

<!--Col Count:8-->Study number	Author (year)	Sample size	Category	Aim	Type of anonymized medical health data	Software and/or algorithm main characteristics	Related outcomes
						Categorical and sensitive attributes protected by improved l-diversity slicing approach	- Classification accuracy ≈ 13% better than the methods Slicing, and Bucketization
41	Pika et al., 2020 Pika et al. (2020)	NA	Data utility Privacy protection	Assessment of data utility and privacy quality	MIMIC dataset, data from intensive care admissions from different hospitals	Anonymization with strategies like data swapping, value suppression, generalization, micro aggregation, and noise addition	- Different levels of performance in various approaches, dependent on the level of required privacy level<br>- Minimal distortion of results by Generalization and small values of k, while Activity suppression affected the results significantly

<!--Col Count:8-->Study number	Author (year)	Sample size	Category	Aim	Type of anonymized medical health data	Software and/or algorithm main characteristics	Related outcomes
							- Privacy protection feasible by the proposed approach, that utilizes privacy metadata
					Data from academic hospital event log		
42	Poulis et al., 2017 Poulis et al. (2017)	200,000 electronic records	De-identification	De-identification of records containing diagnosis codes and demographics	Datasets from university medical center and different hospitals.	ART_{UC}, based on (k, k^m) anonymity, protection from attackers possessing up to m diagnosis codes	- ART_{UC} better than the algorithm RM_R in data utility
							First successful anonymization of medical RT-datasets (protection of diagnosis codes only)

<!--Col Count:8-->Study number	Author (year)	Sample size	Category	Aim	Type of anonymized medical health data	Software and/or algorithm main characteristics	Related outcomes
43	Solomon et al., 2012 Somolinos et al. (2015)	10,865 participants	RR	Assessment of RR in two datasets and simulation of de-identification attacks	Two data sets about sexual health from participant recruited through advertisement	Narayanan and Shmatikov’s algorithm utilized for reidentification assessment	- The higher the diversity of population and uniqueness of a dataset, the higher the risk of reidentification<br>- Combination of 3 attributes creates almost 100% unique records<br>- % of participants reidentified between 93% and 100% in dataset 2
44	Somolinos et al., 2015 Loukides et al. (2010a)	NA	Pseudonymization	Pseudonymization of data for secondary use	Various hospital registry data sets	Clinical and demographic data separated before pseudonymization	- Successful pseudonymization achieved<br>- Possible for researchers to adjust the parameters, thus making pseudonymization

<!--Col Count:8-->Study number	Author (year)	Sample size	Category	Aim	Type of anonymized medical health data	Software and/or algorithm main characteristics	Related outcomes
							most efficient for their requirements
45	Stubbs and Uzuner 2014 Stubbs and Uzuner (2014)	1,304 longitudinal records of 296 patients	Anonymization	Anonymization of longitudinal data	Patients with various diseases	Multi-purpose Annotation<br>Environment used for annotation afterwards relevant surrogates created to replace the original PHI.	- Anonymization of longitudinal achieved for the first time<br>- The maximum and average F-measure 0.964 and 0.872 respectively<br>- Maximum precision, recall, and F1 measure of the new approach higher than inter annotator agreement by 0.062, 0.030, and 0.047 respectively

<!--Col Count:8-->Study number	Author (year)	Sample size	Category	Aim	Type of anonymized medical health data	Software and/or algorithm main characteristics	Related outcomes
						2014 de-identification shared task guidelines applied	- Maximum precision, recall and F1 of the new approach higher than the token based inter annotator agreement by 0.042, 0.019, and 0.033 respectively
46	Sweeney 1998 Sweeney (1998)	300 patients	Anonymization	Anonymization of MHD	Pediatric patients	Anonymization by generalization, insertion, substitution and removing of PHI achieved by Datafly	- The most general form of data for recipient with minimal linking and matching of data provided by Datafly<br>- Possible for Datafly to work with multiple tables in contrast to µ-Argus program

<!--Col Count:8-->Study number	Author (year)	Sample size	Category	Aim	Type of anonymized medical health data	Software and/or algorithm main characteristics	Related outcomes
							- Efficient implementation of the algorithm in large number of demographics in contrast fields in contrast to µ-Argus
47	Tamersoy A et al., 2012 Tamersoy et al. (2012)	NA	Anonymization Data utility	Sharing patient-specific data with robust privacy and high data utility	Patient registry data from University medical data set.	Longitudinal Data Anonymizer algorithm	- Data sharing with high privacy provided for the first time
							- Data with low level of information loss was generated
						Anonymization by implantation of clustering and alignment and treating them as	- Average information loss below 0.5 as measured by normalized loss

<!--Col Count:8-->Study number	Author (year)	Sample size	Category	Aim	Type of anonymized medical health data	Software and/or algorithm main characteristics	Related outcomes
						separate components	
						Preservation of data utility and Alignment using Generalization and Suppression	- Positive correlation between Information loss and k value
48	Templ et al., 2022 Templ et al. (2022)	280,381 events, containing demographic and socioeconomic information	Anonymization	Propose a method for anonymizing event history data	Event history data from Karonga Health and Demographic Surveillance System Core Residency	Sequential noise addition to event dates<br>k-anonymity with local suppression	- Anonymized event history data with high utility was created<br>- Low disclosure risk as calculated by distance-based neighborhood matching<br>- High data utility for low and high levels of noise as

<!--Col Count:8-->Study number	Author (year)	Sample size	Category	Aim	Type of anonymized medical health data	Software and/or algorithm main characteristics	Related outcomes
						R package sdcMicro utilized	assessed by chi-square tests for comparing contingency tables of original and anonymized datasets<br>- The approach limited the number of response categories for time-varying variables
49	Tucker et al., 2016 Tucker et al. (2016)	Not relevant	Data utility Privacy risk	Providence of recommendation for balancing data utility and minimizing privacy risk	Not relevant	General guidelines for creation of anonymized data in accordance with HIPAA rules	- Removing of all HIPAA direct identifiers<br>- Replacement of patient identifier codes with random identifiers<br>- Secure storation of code key

<!--Col Count:8-->Study number	Author (year)	Sample size	Category	Aim	Type of anonymized medical health data	Software and/or algorithm main characteristics	Related outcomes
							- Replacement of dates with age
							- Utilization of secure 'locked box' in data sharing for providence of additional protection
50	Wu et al., 2013 Wu et al. (2013)	-	Data utility	Evaluation of data utility	Adult and IPUMS datasets	Utility assessment of anonymized data by k-anonymity, l-diversity, and t-closeness	- Best overall performance in by k anonymity, generation of the data set with highest utility as measured by precision, recall, F-measure, and accuracy
							- Worst overall performance by t-closeness

<!--Col Count:8-->Study number	Author (year)	Sample size	Category	Aim	Type of anonymized medical health data	Software and/or algorithm main characteristics	Related outcomes
							- Precision from k-anonymity significantly better than l-diversity and t-closeness in both data sets
51	Ye et al., 2011 Ye and Chen (2011)	30,162 records	Anonymization Data utility	Extension of k-anonymity, to meet heterogeneous requirements of various researchers	Adult dataset	Extension of k-anonymity by generalization approach that maintained the consistency of attributes in terms of attribute utility	- Better performance than Incognito and Mondrian
							- Impact of increasing k on data distortion much smaller for AUM compared to the other approaches
						New algorithm: Attribute Utility Motivated k-anonymization (AUM)	- The information loss for AUM decreases linearly with k while that for incognito falls exponentially

<!--Col Count:8-->Study number	Author (year)	Sample size	Category	Aim	Type of anonymized medical health data	Software and/or algorithm main characteristics	Related outcomes
							- AUM exhibits much smaller information loss as measured by Normalized Average Equivalence Class Size Metric
52	Yoo et al., 2012 Yoo et al. (2012)	30,162 patients	De-identification Data utility	Anonymization with low information loss and low diversity of sensitive attributes	Adult dataset with various diseases	k-anonymity combined with l-diversity	- De-identification with minimum information loss and diversity of sensitive attributes achieved
							- Information loss in the proposed method less than l-diversity and conditional entropy
							- Fastest performance time by Entropy l-diversity followed by conditional

<!--Col Count:8-->Study number	Author (year)	Sample size	Category	Aim	Type of anonymized medical health data	Software and/or algorithm main characteristics	Related outcomes
							entropy, t-closeness, and the proposed method
53	Yu and Ji 2014 Yu and Ji (2014)	375 participants	De-identification Data utility	Anonymization of a genomic dataset and assessment of data utility	Data from Personal Genome Project and HapMap	Anonymization by differential privacy the “Laplace mechanism” and “exponential mechanism” of differential privacy applied	- Better utility by exponential mechanism than the other methods for small values of ∈, but significantly faster plateau of exponential mechanism compared to other methods with increasing ∈

Reviews

<!--Col Count:8-->Study number	Author (year)	Number of included studies/algorithms	Category	Aim	Type of anonymized medical health data	Software and/or algorithm main characteristics	Related outcomes
54	Davis and Osoba 2019 [Davis and Osoba (2019)]	-	Anonymization	Analysis of various anonymization approaches	Various datasets	k-anonymity, l-diversity and t-closeness reviewed	- Data utility prioritized over privacy in many cases<br>- More sufficient de-identification by k-anonymity at the cost of higher homogeneity attack risk<br>- l-diversity prone to skewness attack<br>- Higher privacy protection by t-closeness at the cost of greater diminishment of data

<!--Col Count:8-->Study number	Author (year)	Number of included studies/algorithms	Category	Aim	Type of anonymized medical health data	Software and/or algorithm main characteristics	Related outcomes
							utility compared to k-anonymity and l-diversity
55	Gkoulalas-Divanis et al., 2014 Gkoulalas-Divanis et al. (2014)	More than 45 algorithms	Anonymization	Review, strengths, and limitations of different anonymization algorithms assessed	Structured datasets	Various algorithms discussed	- Generalization > suppression in general
							- Data truthfulness possibly harmed by microaggregation
							- High information loss induced by suppression
							- Heuristic strategies', anonymization with minimal loss, Binary, and

<!--Col Count:8-->Study number	Author (year)	Number of included studies/algorithms	Category	Aim	Type of anonymized medical health data	Software and/or algorithm main characteristics	Related outcomes
							Apriori-like lattice search strategies, possibly unable to preserve data utility to the extent of genetic strategies<br><br>- Worst complexity of all heuristic strategies exponential<br><br>- Specific utility requirement not considered by any demographic anonymization algorithm
56	Kolasa et al., 2021 Kolasa et al. (2021)	21 apps reviewed	Anonymization	Capability assessment of COVID-19 contact tracing	Data from users	Systematic review criteria utilized for compliance assessment of	- 48% of apps utilized anonymization techniques

<!--Col Count:8-->Study number	Author (year)	Number of included studies/algorithms	Category	Aim	Type of anonymized medical health data	Software and/or algorithm main characteristics	Related outcomes
			Data utility	apps to balance privacy and data utility		apps with data privacy regulations.	- The best and worst apps to balance privacy protection and data utility: COVIDSafe and Alipay respectively<br>- Majority of apps compliant with data privacy standards
57	Langarizadeh et al., 2018 Langarizadeh et al. (2018)	18 studies	Privacy preservation systematic review.	Assessment of the effectivity of anonymization approaches in preserving privacy	Structured data	Various approaches from different studies included	- Anonymization achievable with current approaches, but RR cannot be eliminated fully

<!--Col Count:8-->Study number	Author (year)	Number of included studies/algorithms	Category	Aim	Type of anonymized medical health data	Software and/or algorithm main characteristics	Related outcomes
							- Secondary use rendered impossible with removal of a large portion of identifiable data<br><br>- An anonymization specifically designed for one type of structured data, not necessarily applicable to other types
58	Olatunji et al., 2021 [Olatunji et al. (2021)]		Anonymization Data utility	Assessment of strengths and limitations of different approaches	Structured data	Many approaches were assessed including but not limited to k-anonymity and	- k-degree-l-diversity protects against node existence attack. The added noise could lead to lower data utility

<!--Col Count:8-->Study number	Author (year)	Number of included studies/algorithms	Category	Aim	Type of anonymized medical health data	Software and/or algorithm main characteristics	Related outcomes
						many variants, t-closeness, l-diversity, utility-aware anonymization, and the LKC privacy model	- t-closeness provides better protection than l-diversity<br>- t-closeness could protect against membership disclosure attacks with the correct t value<br>- Higher t-value could lead to a decrease in data utility<br>- More accurate results were obtained by Global- rather than local differential privacy, only if adequate noise is added to the former method

<!--Col Count:8-->Study number	Author (year)	Number of included studies/algorithms	Category	Aim	Type of anonymized medical health data	Software and/or algorithm main characteristics	Related outcomes
59	Sánchez et al., 2019 Sánchez et al. (2014)	-	Anonymization Data utility	Achieve robust privacy and preserve data utility concurrently	Patient discharge data Hospital clinic data	k-anonymity micro aggregation with t-closeness model nominal values management with semantic mechanisms	- Anonymization tool: μ-ANT highly suitable for large datasets and easily executable software<br>- μ-ANT Higher data utility than ARX and UTD anonymization toolbox
60	Tinabo et al., 2009 Tinabo et al. (2009)	-	Anonymization Pseudonymization	Assessing the risks and benefits of anonymization vs. pseudonymization	Structured data	Review of different anonymization- and pseudonymization algorithms	- Pseudonymization harder to implement and infrequently adopted in contrast to anonymization<br>- Pseudonymization especially useful for de-identification of large datasets

<!--Col Count:8-->Study number	Author (year)	Number of included studies/algorithms	Category	Aim	Type of anonymized medical health data	Software and/or algorithm main characteristics	Related outcomes
61	Zuo et al., 2021 Zuo et al. (2021)	239 studies	Anonymization Risk of reidentification	A systematic review of anonymization approaches	Various datasets	Approaches included but not limited to many variants of k-anonymity, differential privacy, joint differential privacy, k-automorphism, and machine learning approaches such as principal component analysis	- The software Anonimatron performs pseudonymization and is GDPR compliant<br>- ARX had an overall better performance than University of Texas at Dallas Toolbox<br>- High privacy level provided by: (k, e)-, (k, g) anonymity, normalized variance, and (c,t) isolation<br>- Low privacy level provided by: differential privacy, t-closeness, (X,

<!--Col Count:8-->Study number	Author (year)	Number of included studies/algorithms	Category	Aim	Type of anonymized medical health data	Software and/or algorithm main characteristics	Related outcomes
							Y)-anonymity, and δ-presence
							- Machine learning approaches for anonymization were found only suitable for research
							- Secure multiparty computation was more viable than fully homomorphic encryption
							- Anonymization with sufficient data utility and privacy protection is feasible, but requires more research

Books

<!--Col Count:8-->Study number	Author (year)	Sample size	Category	Aim	Type of anonymized medical health data	Software and/or algorithm main characteristics	Related outcomes
62	El Emam & Arbuckle 2014 El Emam and Arbuckle (2014)	Not relevant	Anonymization	A practical guide on anonymization with preservation of the analytical power of data	Cross-Sectional Data (Research Registries)	Risk-Based De-Identification Methodology	- After indicating plausible attack types the authors have adopted aforementioned de-identification techniques to undergo threat modeling and risk assessment for different data sources
					Longitudinal Discharge Abstract Data (State Inpatient Databases)	Following 3 de-identification techniques are adopted throughout: generalization, suppression and subsampling	- The de-identification is generalized and categorized on the basis of 'plausible attack' type

<!--Col Count:8-->Study number	Author (year)	Sample size	Category	Aim	Type of anonymized medical health data	Software and/or algorithm main characteristics	Related outcomes
					Insurance Claims Data Longitudinal Events Data (A Disaster Registry)<br><br>Geospatial Aggregation (Dissemination Areas and ZIP Codes)<br><br>Medical Codes<br><br>Oncology Databases	Other techniques outlined include randomization, pseudonymization, hashing, encryption and shuffling	- Discussed algorithms included in Tables 1 and 2
63	Gkoulalas-Divanis and	Not relevant	Anonymization	A handbook of medical data privacy	Demographics	Differential privacy algorithms	- Review of algorithms utilizing Differential privacy for protection,

<!--Col Count:8-->Study number	Author (year)	Sample size	Category	Aim	Type of anonymized medical health data	Software and/or algorithm main characteristics	Related outcomes
	Loukides 2015 [Gkoulalas-Divanis and Loukides (2015)]						transaction, and stream data protection of medical healthcare data
					Diagnosis codes	Algorithms such as DPCopula and PrivBasis	- An extension of differential privacy, w-Event Privacy, is considered to tend to the needs of infinite real-time streams of medical data
					Sensitive transaction data		

<!--Col Count:8-->Study number	Author (year)	Sample size	Category	Aim	Type of anonymized medical health data	Software and/or algorithm main characteristics	Related outcomes
					Stream data		

The main findings were separated into three parts: primary research articles, reviews, and books.

Abbreviations: ABM-1: accumulated based method, ARE: average relative error, AUM: Attribute Utility Motivated k-anonymization, BTS: blood transfusion service, CBA: clustering based anonymizer, DAD: discharged abstract data, DF: Delay-free Anonymization, FAERS: FDA, adverse event reporting system, GG: greedy grouping, KL-divergence: Kulback-Liebler divergence, LM: loss metric, MCC: Matthews correlation coefficient, MHD: medical health data, NIL: normalized information loss, OLA: optimal lattice anonymization, PHI: personal health information, PRR: proportional reporting ratio, PPDM: privacy preserving data publishing, PUMF: public use microdata file, RE: reconstruction error, RGG: randomized greedy grouping, RR: risk of reidentification, SNB: slide negative binomial, SRLA: sequential rank label anonymization, SRS: spontaneously reporting systems, TFRP: two fixed reference points, UCI: University of California Irvine.
